# Factors Associated with Self-Report of Type 2 Diabetes Mellitus in Adults Seeking Dental Care in a Developing Country

**DOI:** 10.3390/ijerph20010218

**Published:** 2022-12-23

**Authors:** Sandra Aremy López-Gómez, Blanca Silvia González-López, Rogelio José Scougall-Vilchis, María de Lourdes Márquez-Corona, Mirna Minaya-Sánchez, José de Jesús Navarrete-Hernández, Rubén de la Rosa-Santillana, Gladys Remigia Acuña-González, América Patricia Pontigo-Loyola, Juan José Villalobos-Rodelo, Carlo Eduardo Medina-Solís, Gerardo Maupomé

**Affiliations:** 1 School of Behavioral Sciences, Autonomous University of the State of Mexico, Toluca 50130, Mexico; 2 Academic Area of Dentistry of Health Sciences Institute, Autonomous University of Hidalgo State, Pachuca 42160, Mexico; 3 Advanced Studies and Research Center in Dentistry “Dr. Keisaburo Miyata”, Faculty of Dentistry, Autonomous University of the State of Mexico, Toluca 50130, Mexico; 4 School of Dentistry, Autonomous University of Campeche, Campeche 24039, Mexico; 5 School of Dentistry, Autonomous University of Sinaloa, Culiacan 80040, Mexico; 6 Richard M. Fairbanks School of Public Health, Indiana University/Purdue University, Indianapolis, IN 46202, USA

**Keywords:** oral medicine, diabetes, prevalence, epidemiology, adults, dental setting

## Abstract

The aims of the present study were to identify the prevalence and risk indicators of type 2 diabetes mellitus (T2DM) in urban-based Mexican adults seeking care in a university-based triage/intake dental clinic, and to develop a predictive model. A cross-sectional study was conducted on 3354 medical/dental records of adults who sought care at the triage/intake dental clinics of a public university. The dependent variable was self-report of a previous diagnosis of T2DM made by a physician. Several socio-demographic and socioeconomic covariates were included, as well as others related to oral and general health. A multivariate binary logistic regression model was generated. We subsequently calculated well-known statistical measures employed to evaluate discrimination (classification) using an (adjusted) multivariate logistic regression model (goodness-of-fit test). The average age of patients was 42.5 ± 16.1 years old and the majority were female (64.1%). The prevalence of T2DM was 10.7% (95%CI = 9.7–11.8). In the final multivariate model, the variables associated (*p* < 0.05) with the presence of T2DM were older age (40 to 59 years old, OR = 2.00; 60 to 95 years old, OR = 2.78), having any type of health insurance (OR = 2.33), having high blood pressure (OR = 1.70), being obese (OR = 1.41), and having a functional dentition (OR = 0.68). Although the global fit of the model and the calibration tests were adequate, the sensitivity (0.0%) and positive predictive (0.0%) values were not. The specificity (100%) and negative predictive (89.3%) values, as well as the correctly classified (89.3%) value, were adequate. The area under the ROC curve, close to 0.70, was modest. In conclusion, a prevalence of T2DM of 10.7% in this sample of Mexican adults seeking dental care was similar to national figures. Clinical (blood pressure, BMI and functional dentition), demographic (age), and socioeconomic (health insurance) variables were found to be associated with T2DM. The dental setting could be appropriate for implementing preventive actions focused on identifying and helping to reduce the burden of T2DM in the population.

## 1. Introduction

Among the non-communicable diseases of high prevalence in the population are cardiovascular diseases, different types of cancer, chronic respiratory diseases, and diabetes mellitus. Diabetes includes a group of metabolic diseases characterized by chronic hyperglycemia due to a deficit in insulin secretion, deficient insulin action, or both. Diabetes mellitus is a global public health problem due to it is prevalence, associated morbidity, and high mortality risk. A variety of complications of varying importance have been demonstrated in patients with long-standing diabetes [1,2]. In general, diabetes is the result of a combination of genetic, physiological, environmental, and behavioral factors [3,4].

In recent decades, the prevalence of diabetes and its complications has increased drastically, to the point of reaching epidemic proportions [5]. Mexico is in the top ten of countries in terms of the number of people who have diabetes in the years 2021 (seventh) or forecasted for 2045 (eighth) [6]. Survey data show an increase in its prevalence [7,8,9]. In Mexico, it is estimated that by 2030 the prevalence will reach 12–18% and, by 2050, 14–22% [10]. In general, type 2 diabetes mellitus (T2DM) is the result of a combination of genetic, physiological, environmental, and behavioral factors. Among the factors that determine its high prevalence are aging of the population, increase in obesity associated with high dietary caloric density, and low physical activity [11]. Socio-demographic factors may also be implicated (sex, religion, marital status, school level, occupation, and income) [12,13]. Access to health services for chronic disease care [14], lifestyle management, glucose control [15,16], blood pressure, and cholesterol levels in populations with poor socioeconomic conditions [17] contribute to a delay in the diagnosis of chronic diseases in general, and to the delay in detecting complications associated with T2DM [18].

Knowing whether a patient has T2DM or could be at risk is an important piece of clinical information in dental practice. Introducing such concept in dental education helps students to frame dental care and diagnoses within the larger landscape of systemic diseases. Moreover, greater awareness about T2DM and its frequency offer opportunities for holistic care, for health promotion interventions in patient waiting areas, or, in the context of dental care at large [19,20], making clear the value of an inter-professional, team-based approach to the prevention and treatment of patients at risk or affected by one or both conditions. Dental care settings may contribute to T2DM care as well as in its identification [21,22]. The aims of the present study were to identify the prevalence and risk indicators of T2DM in urban-based Mexican adults seeking care in a university-based triage/intake dental clinic, and to develop a predictive model.

## 2. Materials and Methods

### 2.1. Design and Place of the Study

An analytical cross-sectional study was conducted on a random sample of medical records of patients who sought dental care at the Dentistry Academic Area of the Autonomous University of the State of Hidalgo, in Mexico. Part of the methodology has been previously published [23,24]. The following criteria were used to calculate the sample size: the universe was 16,500 medical records, the heterogeneity (diversity of the universe) was 50%, the margin of error was 2%, and a confidence level of 99; a sample of 3315 was obtained. A 5% was added to account for missing values, so the final sample was 3481 medical records. The inclusion criteria were medical records of individuals of (1) either sex, (2) 18 years of age or older, and (3) having been diagnosed with type 2 diabetes mellitus. The exclusion criteria were (1) incomplete medical records and (2) medical records unavailable at the time of the study. After applying the inclusion and exclusion criteria, the final sample was 3354.

### 2.2. Data Collection

The medical/dental records analyzed in the present study were filled out by senior dental students in their 10th semester (5th year) of the dental program. The students who participated in the data collection were trained and the criteria standardized.

### 2.3. Study Variables

The dependent variable was self-report of having received a diagnosis of T2DM by a physician, which was dichotomized as 0 = no and 1 = yes. The independent variables were age (18–39; 40–59; 60–95); sex (female, male); socioeconomic status (measured through occupation as: low, medium, high; and type of housing as: good, fair, poor); diet (good, fair, poor); marital status (married, divorced, single, cohabitation, widowed); health insurance (Uninsured, With insurance, as contemplated by carriers available in Mexico [Mexican Institute of Social Security; Institute of Security and Social Services for State Workers, other institution, Seguro Popular]); blood pressure [normal, high, stage 1 hypertension (HT1), stage 2 hypertension (HT2)]; body mass index (BMI) (underweight, normal, overweight, obese); and functional dentition (No, Yes).

Occupation was classified according to the National Occupational Classification System and then categorized as level low, medium, and high [25]. The variable housing type was classified according to the type of material used for the walls, floor, and roof of the house, as well as the availability of water and sewage. Diet was self-reported by the individuals, according to what they considered a balanced consumption of meat proteins, fruits and vegetables, carbohydrates derived from flour, sugars and fats. Blood pressure was classified according to American College of Cardiology/American Heart Association Task Force on Clinical Practice Guidelines [26] [Hypertensive crisis (systolic blood pressure (SBP) > 180 mmHg or diastolic BP (DBP) > 120 mmHg; Elevated (SBP 120–129 mmHg and DBP < 80 mmHg); Hypertension 1 (SBP 130–139 mmHg or DBP 80–89 mmHg); Hypertension 2 (SBP ≥ 140 mmHg or DBP ≥ 90 mmHg); and Normal (SBP < 120 mmHg and DBP < 80 mmHg)]. Body Mass Index (BMI) followed the Centers for Disease Control and Prevention [27] description, weight in kilograms divided by squared height in meters. A high BMI may indicate excess weight. The number of missing teeth was counted and classified as functional dentition. It was scored with 0 indicating subjects with fewer than 21 teeth present in the mouth and 1, indicating subjects with 21 or more teeth [28].

### 2.4. Statistical Analysis

Univariate analysis used measures of central tendency and dispersion for continuous variables, and frequencies and percentages for categorical variables.

For bivariate and multivariate analyses, binary logistic regression models were used. The strength of the association between the dependent variable (T2DM) and the independent variables was expressed as odds ratio (OR) with 95% confidence intervals (95%CI). A value of *p* < 0.05 was considered statistically significant. The variance inflation factor (VIF) test was performed to analyze and, if necessary, avoid multicollinearity between the independent variables. For the construction of the model, those variables that in the bivariate analysis showed a value of *p* < 0.25 were incorporated. The overall fit of the model was performed with the goodness-of-fit test [29].

Using an (adjusted) multivariate logistic regression model (goodness-of-fit test), we calculated statistical measures [30] used to evaluate discrimination (classification) performance of diagnostic tests: sensitivity (true positive rate) as a measure of the proportion of compatible correctly identified; specificity (true negative rate) as a measure of the proportion of correctly identified; the false positive rate as a measure of the proportion of comparisons incorrectly reported as compatible when they were actually incompatible; false negative rate as a measure of the proportion of comparisons incorrectly reported as incompatible when they were actually compatible; and the receiver operating characteristic (ROC) curve, with the area under the ROC curve (AUC).

For the validation analysis, we followed an approach to develop a model for predicting diabetes through dividing our data in two sub-groups, a development sample and a validation sample. We randomly separated the T2DM data in the two samples. Then, we conducted the test in one sample: to test calibration in the developmental sample, we calculated the goodness-of-fit test. Then, we tested the calibration of our model by performing a goodness-of-fit test on the other (validation sample), followed by the tests incorporated in the original model.

The Stata statistical package version 14 was used for all analyses (Copyright StataCorp LP; College Station, TX 77845, USA).

### 2.5. Ethics Statement

The project was approved by the ethics and research committee of the Institute of Health Sciences of the Ethics and Research Committee of the Institute of Health Sciences of the Autonomous University of the State of Hidalgo (CEEI-000036-2019).

## 3. Results

A total of 3354 individuals were included in the study. The average age was 42.5 ± 16.1 years old, 42.7% were between 18 and 39 years of age, and the majority were female (64.1%). The description of independent variables is in Table 1. The prevalence of T2DM was 10.7% (95%CI = 9.7–11.8) (n = 359).

The prevalence of T2DM across the categories of independent variables is shown in Table 2, including the bivariate logistic regression analysis. Older age, marital status (married and widowed), having some type of health insurance, self-reported poorer diet quality, high blood pressure, high BMI (obesity), and functional dentition were found to be significant (*p* < 0.05). Sex, occupation, and housing were not found to be associated with the presence of T2DM.

In the final binary logistic regression model shown in Table 3, older people [40–59 years old, OR = 2.00 (95%CI = 1.51–2.65); 60–95 years old OR = 2.78 (95%CI = 1.92–4.01)] had higher odds of having received a T2DM diagnosis. People with any type of health insurance (OR = 2.33, 95%CI = 1.24–4.34) were more likely to have a T2DM diagnosis than those without. People with stage 2 hypertension were more likely (OR = 1.70, 95%CI = 1.21–2.39) to report T2DM than those with normal blood pressure. Among obese people, the likelihood of reporting T2DM increased 41% (95%CI = 5–88%) compared to people with normal BMI. Those with functional dentition were less likely to report diabetes (OR = 0.68, 95%CI = 0.51–0.90) than those without functional dentition.

A logistic regression model (also known as *logit* model) is often used for predictive purposes. The model estimates the likelihood that an outcome would occur (e.g., presence of T2DM) based on a data assembly derived from independent variables. Since the result is a probability, it ranges from 0 to 1 (or a 0% to 100% likelihood the outcome will occur). In such model, probabilities are *logit* transformed, whereby the likelihood of the outcome presenting (success) is divided by the likelihood of the outcome not occurring (failure). This construct is also known as logarithmic probabilities, or natural logarithm of probabilities, commonly synthesized in the following formulae [31]:

*Logit*(pi) = 1/(1 + exp(−pi)) (1)

ln(pi/(1 − pi)) = β_0_ + β_1_X_1_ + … + β_k_X_Ki_ + Є_i_ (2)

*logit*(pi) is the dependent or response variable and X is the independent variable. For the present analysis, the T2DM predictive model is represented as follows:

P(y = Diabetes) = 1/(exp(−3.5457 + 0.6947X_1_ + 1.0236X_2_ + 0.8460X_3_ + 0.5362X_4_ + 0.3465X_5_ − 0.3826X_6_ (3)

where −3.5457 is a constant; X_1_ = 40 to 59 years old; X_2_ = 60 to 95 years old; X_3_ = With insurance; X_4_ = HT2; X_5_ = Obese; and X_6_ = With functional dentition.

**Figure 1:**
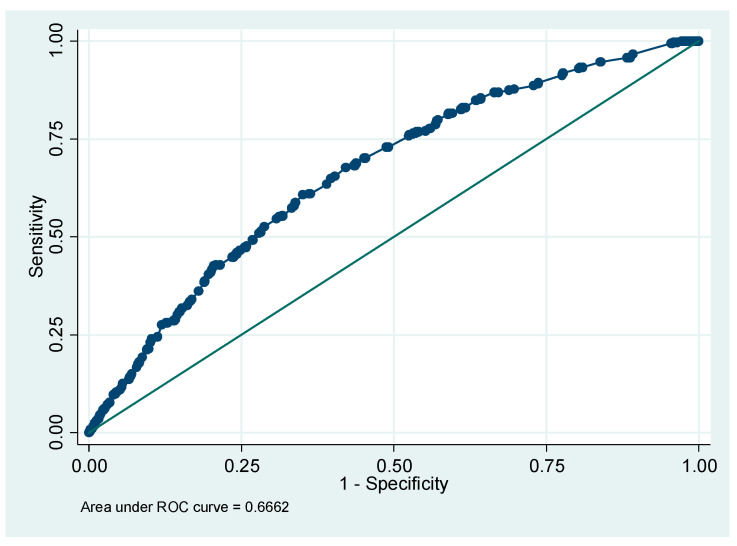
Graph area under ROC curve. Number of observations = 3354. Area under ROC curve = 0.6662.

**Figure 2:**
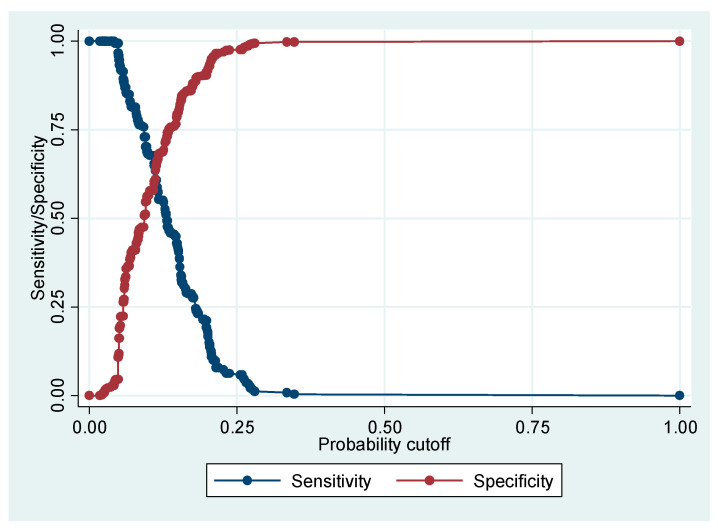
Graph sensitivity and specificity versus probability cutoff.

We present the ROC curve in Figure 1. In Figure 2, we have the sensitivity and specificity values against probability cut-off for the multivariate logistic regression model.

### Diagnostic Test Values for Final Multivariate Model

After the model has been computed, it is best practice to evaluate the how well the model predicts the dependent variable, which is called a Goodness-of-fit test. Since the *p*-value was 0.7929 > 0.05 we know that there were no statistically significant differences between the numbers observed and those predicted.

We undertook a calibration of the model, whereby we randomly allocated the overall sample in two groups (developmental sample and validation sample). Then, we fitted a model using one group (developmental sample) and calculated the goodness-of-fit test. Table 4 shows the results for both groups (developmental sample and validation sample). Our model fitted well the validation sample. The model’s discrimination for the validation sample was *p* > 0.05.

Although the global fit of the model and the calibration tests were adequate, the sensitivity (0.0%) and positive predictive (0.0%) values were not. The specificity (100%) and negative predictive (89.3%) values, as well as the correctly classified (89.3%) value were adequate. The area under the ROC curve, close to 0.70, was modest.

## 4. Discussion

The present study determined the prevalence of diabetes and risk indicators in a large, multiyear sample of adults seeking care at a dental school in a public university in Mexico. There was a relationship between diabetes and certain clinical, socioeconomic, and socio-demographic variables. T2DM prevalence was 10.7%, a slightly higher percentage compared to the most recent National Health and Nutrition Survey in Mexico of 2018 (federal survey with national representativeness), which reported a prevalence of 10.3% [8]. According to estimates for the prevalence of diagnosed T2DM from national health surveys in Mexico, by 2030 the prevalence will increase to 12–18% and by 2050, to 14–22% [10]. These figures will have repercussions for the entire Mexican health system [32]. They will also have an impact on people’s oral health if we consider that individuals with T2DM tend to present negative oral manifestations [33,34], thereby increasing the burden of oral disease and expenditure.

An increase in the prevalence of T2DM with older age was found in the present study; the highest prevalence of T2DM pertained to the 60–95 age group, coinciding with previous studies in Mexico, where 14.4% of adults older than 20 were found to have the disease, with the highest percentage (30%) after age of 50 [35]. This may be due to a combination of two scenarios; on the one hand, older age affects the body irreversibly, from molecules to physiological systems, causing a greater predisposition to chronic/degenerative diseases [36]. On the other hand, the increase in life expectancy among Mexicans (from 50.7 years in 1950 to 75.1 years in 2020) may be happening simultaneously with changes in lifestyle, such as in diet and physical activity [37,38]. Longer life expectancy could accrue higher likelihood of developing T2DM.

Obesity was associated with T2DM, which is consistent with the literature where obese people are at increased risk of having T2DM [11,38,39]. High BMI has been shown to be an important factor for a substantial proportion of the burden of T2DM due to abnormal concentrations of cholesterol, triglycerides, HDL cholesterol, and uric acid [40]. According to the Global Burden of Diseases in 2017, 36.5% of deaths from T2DM worldwide are attributed to high BMI, and in Mexico that percentage rose to 51.8% [40]. In Mexico, the prevalence of overweight and obesity has risen in recent years; from 1980 to date, these have tripled and now more than 70% of the Mexican adult population has excess weight. Such association between T2DM and excess weight seems mainly due to obesity producing a progressive defect in insulin secretion and resistance due to the increase in visceral and ectopic fat deposition; those traits may be present together with a greater infiltration of inflammatory cells that causes a chronic inflammatory state in muscle and liver tissue [41]. A similar association was found with hypertension; its association with T2DM coincides with other studies [42,43] where hypertensive individuals were 3.5 times more likely to be diabetic. Various epidemiological studies showed that a number of cases of arterial hypertension may be explained by excess adipose tissue in obese individuals [44]. Arterial hypertension is an extremely frequent comorbidity in diabetics, with a prevalence of hypertension 1.5 to 3 times higher than in non-diabetics. Hypertension also contributes to the development and progression of chronic complications of T2DM [45].

Having some type of health insurance was associated with the experience of T2DM. This health insurance variable is often used in the Mexican environment as a proxy for socioeconomic status, so that people with better socioeconomic status (those with some type of health insurance) were more likely to have T2DM. This could be associated with greater affluence and a richer diet. However, it could be reflecting that people with health insurance have more and better access to T2DM screening than their less advantaged peers. This could be masking the true prevalence among those who do not have health insurance and who may be affected, but unaware or had not yet been diagnosed by a physician. According to data from the National Council for the Evaluation of Social Development Policy, in Mexico approximately 71.7 million people do not have access to health insurance: 57.3% of the total population. Such feature is more common among people living in any level of poverty [46,47]. Socioeconomic factors and lacking health insurance modify access to and utilization of health services in Mexico [48,49].

Various studies have reported that the condition of the teeth has an impact on morbidity and mortality. According to the literature, it is known that only one in three developing countries have generally available basic technologies for diagnosis and comprehensive care [50]. Dental offices may be a setting for the detection, or help in the control of, T2DM. In the present study, lack of functional dentition (21 or more natural teeth in the mouth) was identified as a T2DM risk indicator (protective factor). That is, when a significant number of teeth are present in the mouth, a clinician could subjectively presume a lower likelihood of T2DM.

We used a Goodness-of-fit test for the T2DM predictive model. Since the *p*-value was 0.7929 > 0.05 we know that there were no statistically significant differences between the numbers observed and those predicted. In essence, the fit is good when the predicted probability is closely associated with Result 1 (T2DM diagnosis) of the dependent variable. Based on the chi-squared test, we were able to contrast the hypothesis that the frequency of observed numbers was the same as those predicted. Although the approach (like most frequency statistics) may be distorted by large sample sizes, this was not the case for our model. Such validation is an important part of the predictive model and its development: it determines reproducibility of the predictive model and avoids over-interpretation of actual data. Logistic regression models can be used to ascertain risk factors (explanatory model) or to predict whether an outcome will occur (predictive model) [51]; we presented both in the present analysis. Above and beyond the identification of factors associated with T2DM and the robust validation and adjustment, we emphasize the need to consider cautiously the results from the predictive model; such note is warranted because some of the test values were not ideal.

The results of the present study have public health and clinical implications. On the one hand, it is an opportunity to take action to diagnose, prevent, and reduce the burden of T2DM consequences. On the other hand, dentists should be aware that a significant proportion of their patients may have T2DM and consider this factor in clinical dental care. In addition, a significant percentage of patients may not have a diagnosis of T2DM: a national study found a 4.1% higher prevalence if undiagnosed cases were added [52].

Among the limitations of the study, we have its cross-sectional nature, where cause and effect are measured at the same time; it is not possible to establish relationships beyond statistical associations. Another limitation could be due to the setting in which the study was conducted, which was within a captive sample seeking dental care. This may have introduced selection bias; the study population is not representative of the general population. Levels of glycemic control are related to the severity of diabetic complications; not having such data available for our study may have affected the results. Self-report of diabetes by patients might not be accurate; patients might have a misconception that their diabetes is “cured” and therefore they no longer would report it, or even not knowing they have diabetes. Therefore, T2DM self-report may have led to a prevalence underestimation.

## 5. Conclusions

In conclusion, a prevalence of T2DM of 10.7% in this sample of Mexican adults seeking dental care is similar to those national figures. Clinical (blood pressure, BMI and functional dentition), demographic (age), and socioeconomic (health insurance) variables were found to be associated with T2DM. Such higher prevalence brings special attention to the implications when caring for dental patients, e.g., offering a dedicated space to implement preventive actions focused on reducing the burden of T2DM in this population, as well as considering a potential diabetes diagnosis in planning and delivering dental care.

Although the global fit of the model and the calibration tests were adequate, the sensitivity (0.0%) and positive predictive (0.0%) values were not. The specificity (100%) and negative predictive (89.3%) values, as well as the correctly classified (89.3%) value were adequate. The area under the ROC curve, close to 0.70, was modest. The value of the model should be cautiously interpreted.

The dental setting could be appropriate for implementing preventive actions focused on identifying and helping to reduce the burden of T2DM in the population. Overall diabetes care could then be strengthened by adding dental settings to the implementation of T2DM prevention and health promotion protocols. Dentists could serve as sentinels for T2DM risk as part of the multidisciplinary health team, while at the same time reminding the patient about the importance of oral health in diabetes control and vice versa.

## Figures and Tables

**Table 1:** Descriptive characteristics of the study sample.

Variable	Average	SD
Age	42.5	16.1
	Frequency	Percentage
Age 18 to 39 years old 40 to 59 years old 60 to 95 years old	1433 1403 518	42.7 41.8 15.5
Sex Female Male	2151 1203	64.1 35.9
Occupation Low Medium High	2943 194 217	87.7 5.8 6.5
Marital status Married Divorced Single Cohabitation Widowed	1438 91 1351 324 150	42.9 2.7 40.3 9.6 4.5
Health insurance Uninsured With insurance	215 3139	6.4 93.6
Housing Good Fair Poor	2156 1146 52	64.3 34.2 1.5
Diet Good Fair Poor	1231 1857 266	36.7 55.4 7.9
Blood pressure Normal High HT1 HT2	1460 291 1227 376	43.5 8.7 36.6 11.2
BMI Underweight Normal Overweight Obese	124 1244 1232 754	3.7 37.1 36.7 22.5
Functional dentition No Yes	677 2677	20.2 79.8

**Table 2:** Bivariate logistic regression analysis for diabetes and independent variables.

Variable	Prevalence	OR (95%CI)	*p*-Value
Age 18 to 39 years old 40 to 59 years old 60 to 95 years old	5.9 12.5 19.1	1 * 2.26 (1.72–2.96) 3.74 (2.74–5.10)	<0.001 <0.001
Sex Female Male	10.4 11.3	1 * 1.10 (0.87–1.38)	0.400
Occupation Low Medium High	10.4 13.9 12.4	1 * 1.39 (0.91–2.13) 1.22 (0.80–1.87)	0.121 0.336
Marital status Married Divorced Single Cohabitation Widowed	12.2 13.2 8.6 9.6 16.0	1.48 (1.15–1.90) 1.61 (0.85–3.05) 1 * 1.12 (0.74–1.70) 2.02 (1.25–3.26)	0.002 0.139 0.575 0.004
Health insurance Uninsured With insurance	5.1 11.1	1 * 2.31 (1.24–4.28)	0.008
Housing Good Fair Poor	11.2 10.0 5.8	2.05 (0.63–6.64) 1.82 (0.55–5.93) 1 *	0.229 0.320
Diet Good Fair Poor	9.7 10.9 13.9	1 * 1.14 (0.90–1.45) 1.50 (1.01–2.24)	0.260 0.041
Blood pressure Normal High HT1 HT2	9.0 11.0 11.1 15.7	1 * 1.24 (0.82–1.87) 1.25 (0.97–1.61) 1.87 (1.34–2.60)	0.297 0.079 <0.001
BMI Underweight Normal Overweight Obese	8.1 9.2 10.5 13.9	0.86 (0.43–1.69) 1 * 1.14 (0.88–1.49) 1.58 (1.19–2.10)	0.664 0.306 0.001
Functional dentition No Yes	18.3 8.8	1 * 0.42 (0.33–0.54)	<0.001

* = Reference category.

**Table 3:** Multivariate logistic regression analysis for diabetes and independent variables included in the study.

Variable	OR (95%CI)	*p*-Value
Age 18 to 39 years old 40 to 59 years old 60 to 95 years old	1 * 2.00 (1.51–2.65) 2.78 (1.92–4.01)	˂0.001 ˂0.001
Health insurance Uninsured With insurance	1 * 2.33 (1.24–4.34)	0.008
Blood pressure Normal High HT1 HT2	1 * 1.20 (0.79–1.82) 1.18 (0.91–1.53) 1.70 (1.21–2.39)	0.382 0.186 0.002
BMI Underweight Normal Overweight Obese	0.84 (0.42–1.68) 1 * 1.03 (0.79–1.35) 1.41 (1.05–1.88)	0.636 0.794 0.019
Functional dentition No Yes	1 * 0.68 (0.51–0.90)	0.008

* = Reference category. Model adjusted for the variables contained in the table, plus sex. Goodness-of-fit test: Pearson chi^2^ (216) = 198.84, *p* = 0.7929.

**Table 4:** Results from calibration tests in the logistic regression models.

Developmental Sample	Validation Sample
Number of observations = 1677 Number of covariate patterns = 195 Pearson chi^2^ (183) = 148.94 Prob > chi^2^ = 0.9692 Area under ROC curve = 0.6711	Number of observations = 1677 Number of covariate patterns = 191 Pearson chi^2^ (183) = 200.65 Prob > chi^2^ = 0.1280 Area under ROC curve = 0.6792

## Data Availability

The datasets generated during and/or analyzed during the current study are available from the corresponding author on reasonable request.
